# Advancements in Intelligent Sensing Technologies for Food Safety Detection

**DOI:** 10.34133/research.0713

**Published:** 2025-06-02

**Authors:** Wenhui Jiang, Changhong Liu, Wei Liu, Lei Zheng

**Affiliations:** ^1^ Engineering Research Center of Bio-Process, Ministry of Education, School of Food and Biological Engineering, Hefei University of Technology, Hefei 230009, China.; ^2^ Intelligent Control and Compute Vision Lab, Hefei University, Hefei 230601, China.

## Abstract

As a critical global public health concern, food safety has prompted substantial strategic advancements in detection technologies to safeguard human health. Integrated intelligent sensing systems, incorporating advanced information perception and computational intelligence, have emerged as rapid, user-friendly, and cost-effective solutions through the synergy of multisource sensors and smart computing. This review systematically examines the fundamental principles of intelligent sensing technologies, including optical, electrochemical, machine olfaction, and machine gustatory systems, along with their practical applications in detecting microbial, chemical, and physical hazards in food products. The review analyzes the current state and future development trends of intelligent perception from 3 core aspects: sensing technology, signal processing, and modeling algorithms. Driven by technologies such as machine learning and blockchain, intelligent sensing technology can ensure food safety throughout all stages of food processing, storage, and transportation, and provide support for the traceability and authenticity identification of food. It also presents current challenges and development trends associated with intelligent sensing technologies in food safety, including novel sensing materials, edge-cloud computing frameworks, and the co-design of energy-efficient algorithms with hardware architectures. Overall, by addressing current limitations and harnessing emerging innovations, intelligent sensing technologies are poised to establish a more resilient, transparent, and proactive framework for safeguarding food safety across global supply chains.

## Introduction

In the global food industry, safeguarding food safety is of paramount importance as it has direct implications for public health and corporate reputation. Recently, there has been a notable increase in the frequency of food safety incidents globally. These incidents are primarily caused by various contaminants, such as pesticides, veterinary drugs, heavy metals, mycotoxins, pathogens, and the misuse of food additives. To ensure food remains safe for consumption, researchers are committed to developing sensitive and rapid analytical methods for efficient contamination detection. This ongoing research is crucial for preempting potential health risks and upholding the integrity of global food safety standards.

Traditionally, the acquisition of food safety information has largely relied on specific analytical detection techniques. High-performance liquid chromatography (HPLC) and liquid chromatography–mass spectrometry (LC-MS) are employed for the separation and quantitative analysis of compounds, while gas chromatography (GC) and gas chromatography-mass spectrometry (GC-MS) are utilized for the analysis of volatile substances. Additionally, bioanalytical methods such as enzyme-linked immunosorbent assay, which measures antigen–antibody interactions, and polymerase chain reaction, which amplifies DNA sequences, are also employed. These methods are widely used in food safety detection due to their high sensitivity and accuracy. However, they typically require complex sample preparation and skilled technicians, making them unsuitable for rapid analysis, particularly for real-time quality monitoring in the food industry [1]. Moreover, these methods are often limited to sampling or retrospective third-party testing and fail to meet consumer and processor demands for high-quality food.

Intelligent perception technology is the ability to be aware of and learn from experiences, which was originally defined by Keith M. Kendrick in 1998 as the adaptive learning responses animals exhibit to their environment or themselves [2]. As for human intelligent technology, intelligent perception refers to the process in which various advanced sensors, intelligent algorithms, and data processing technologies are utilized to enable devices or systems to automatically acquire, analyze, and understand the information from the surrounding environment, so as to make intelligent decisions and responses [3]. In the context of the food industry, intelligent perception technology can be understood as the integration of traditional sensing techniques with artificial intelligence to simulate certain human perceptual and computational functions in specific scenarios. This enables technology to provide optimal decision-making information for human production and life. Compared to conventional detection methods, intelligent perception technology offers several advantages, including nondestructiveness, high precision, real-time processing, and comprehension ability. It can meet the requirements for online processing in terms of recognition speed and provide comprehensive knowledge of detection targets. The common intelligent sensing technologies include optical, electrical, acoustic, magnetic, machine olfactory, and machine gustatory detection. These technologies are not only highly versatile but also user-friendly, allowing nonprofessionals to operate them and thus reaching a wider audience than traditional methods. This accessibility further enhances the practicality and applicability of intelligent perception technology in various fields. It can be integrated with other technologies such as storage systems, production processes, and blockchain to address a wide range of issues across the food industry. This multifunctional capability highlights its potential as a versatile tool for modern food production and management.

This review investigates the development and application of common intelligent sensing technologies in food safety fields. In addition, it delves into the challenges these technologies encounter throughout the food supply chain and proposes potential solutions to address these issues. Moreover, this review explores future trends in intelligent sensing technologies, emphasizing their growing role in advancing food safety standards and operational efficiency within the industry. This analysis not only underscores the current capabilities and limitations of these technologies but also projects their potential for future impact on global food safety practices.

## Overview of Intelligent Sensing Technology

### Detection technologies in intelligent sensing

#### Optical detection technology

**Fig. 1.**
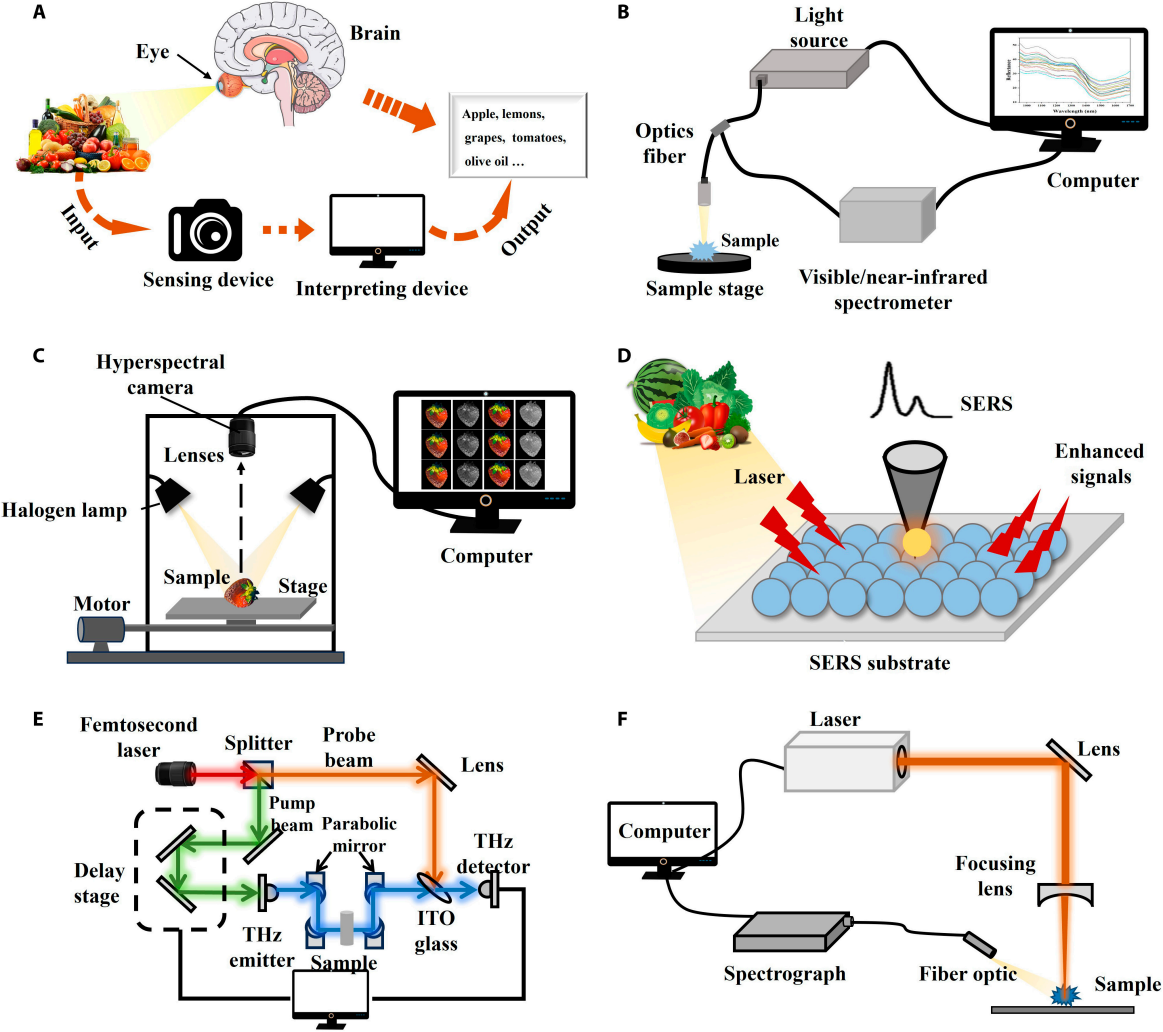
Common optical detection technologies. (A) Machine vision (MV): A camera captures the image and provides it to the computer, which automatically performs tasks that could be done by the human visual system. (B) Near-infrared spectroscopy (NIRS): The technique detects characteristic absorption of 780 to 2,500 nm near-infrared light by C-H, O-H, and N-H groups in molecular structures, combined with chemometric modeling. (C) Hyperspectral imaging (HSI): The technique that simultaneously captures spatial and spectral information, forming a 3D data cube (*x*, *y*, *λ*) for analysis. (D) Surface-enhanced Raman spectroscopy (SERS): A spectroscopic technique that enhances the Raman signal of molecules through metallic nanostructures, achieving single-molecule level sensitivity. (E) Terahertz spectroscopy (THz): A nondestructive technique for characterizing low-frequency vibrational modes of materials by detecting absorption, reflection, or emission of electromagnetic waves across the microwave to infrared range. (F) Laser-induced breakdown spectroscopy (LIBS): A technique for rapid multielement detection by analyzing the emission spectrum of laser-induced plasma generated on the sample surface using pulsed lasers.

Optical detection technologies, which have the characteristics of being noninvasive, rapid, and accurate, are extensively applied in food manufacturing and product testing processes. They utilize light properties such as absorption, scattering, emission, and refraction. In addition, they use models trained using algorithms such as artificial neural networks (ANNs) to analyze food freshness, purity, and safety. Common optical detection technologies include machine vision (MV), near-infrared spectroscopy (NIRS), hyperspectral imaging (HSI), surface-enhanced Raman spectroscopy (SERS), terahertz spectroscopy (THz), and laser-induced breakdown spectroscopy (LIBS). Figure 1 illustrates these techniques schematically.

MV has gained considerable attention in the food industry since its inception in the 1960s [4]. Fueled by enhancements in computational capabilities and algorithmic innovations, MV has undergone remarkable development and has been widely applied in automated inspection tasks. MV systems employ cameras and computers to replace human eyes for recognizing, tracking, and measuring objects, followed by further image processing (Fig. 1A). Through image segmentation, feature extraction, content analysis, and object recognition, raw images are transformed into meaningful datasets from which key assessment indicators are derived. As the MV system foundation, image acquisition typically employs RGB color cameras. These cameras capture images using 3 filters centered on red (R), green (G), and blue (B) wavelengths. Precise measurements require high-resolution cameras and quality lenses. Preprocessing steps, such as noise removal, contrast enhancement, and grayscale conversion, enhance image quality. Effective image segmentation, based on different features, is crucial for feature expression and content analysis. These features feed machine learning algorithms for target recognition [5]. The application of MV in food safety inspection is extremely extensive, covering the entire process of quality control from raw material selection to finished product packaging. Rong et al. [6] captured 1,264 images of juglans using a high-resolution camera and employed a 2-stage convolutional network method to achieve image segmentation and impurity detection, with an accuracy rate of 96.5% and a processing time of less than 60 ms, markedly enhancing both efficiency and accuracy. Moreover, the combination of MV with machine learning algorithms has been instrumental in detecting package completion, thereby reducing associated production costs [7]. Nevertheless, practical MV applications face limitations. Environmental factors, especially fluctuations in lighting and changes in background, can notably reduce detection accuracy. Moreover, the complex shapes, diverse colors, and varying surface textures of food products also make image segmentation and feature extraction more challenging. Often, the integration of high-quality hardware and advanced deep learning algorithms, which can be expensive and intricate, is necessary to overcome these obstacles [8]. It is possible to enhance the adaptability of MV to environmental conditions by utilizing adaptive algorithms and integrating advanced lighting systems [9]. MV integration with complementary sensing technologies may enable more comprehensive, accurate detection [1].

NIRS analyzes molecular vibrations through characteristic absorption spectra (Fig. 1B). When near-infrared (NIR) light interacts with a substance, hydrogen-containing functional groups in organic molecules (e.g., C-H, N-H, and O-H) experience vibrational transitions. These transitions alter the molecular dipole moments, resulting in the absorption of NIR light at specific frequencies and producing absorption spectra within the NIR region of 780 to 2,500 nm (wavenumbers 12,500 to 4,000 cm^−1^) [10]. NIRS is widely adopted in food safety due to its rapid, nondestructive, accurate, cost-effective nature and operational simplicity. For instance, the combination of NIRS and partial least squares discriminant analysis (PLS-DA) has effectively distinguished between different brands of liquor with identical flavor profiles and alcohol content, achieving a high predictive performance with a recognition rate of 98.7% [11]. Carbas et al. [12] formulated partial least squares (PLS) and ANN prediction models using NIRS combined with machine learning algorithms for the quantitative detection of fumonisins. This enhanced fumonisin detection efficiency/accuracy, improving corn supply chain safety monitoring. Unlike traditional methods, NIRS eliminates the need for chemical reagents, thus cutting costs related to waste disposal [13]. In operational settings, the implementation of real-time monitoring through NIRS facilitates the quality and safety assessment of the food supply chain [14]. Despite its advantages, NIRS faces challenges. For trace hazardous substances outside the detection capabilities of NIRS, signal enhancement may be achieved by extending the optical path length or by increasing the number of scans [15]. Concurrently, enriching target analytes during sample preparation can enhance detection sensitivity. Typically, the performance of NIRS is compromised by matrix effects, such as moisture, necessitating the application of spectral preprocessing or wavelength selection techniques to enhance analytical precision [16]. The NIR spectral region is characterized by broad molecular vibration bands and overlapping signals, complicating direct component analysis. Consequently, chemometric methods are employed to develop calibration models, using statistical correlations to assist in peak identification [17]. These methods can be integrated with other spectroscopic technologies to better interpret NIR spectra, thereby enhancing the interpretability of the results [18–20]. On the hardware front, advancements are directed toward miniaturization and integration, which lowers costs and boosts portability, thus enabling immediate on-site detection [21,22].

HSI combines spectroscopy and imaging to provide simultaneous spectral and spatial data. Unlike NIRS, which primarily captures spectral data, HSI additionally analyzes spatial surface characteristics. Moreover, HSI covers a broad spectral range, from ultraviolet to terahertz wavelengths, primarily utilizing the visible (Vis) and NIR bands. This dual capability enables precise physicochemical characterization with high reliability. An HSI system includes essential components such as an array camera, spectroscopic equipment, light sources, transmission mechanisms, and computer hardware and software (Fig. 1C). HSI can be categorized into point, line, or area scanning, depending on the image acquisition and formation methods [23]. Hyperspectral data acquisition involves reflection, transmission, and diffuse transmission methods, each characterizing a unique interaction between light and the target object. These interactions modify light properties, embedding them with a wealth of internal and external information of the object. By analyzing these encoded spectral data, HSI achieves a rapid and nondestructive assessment of critical parameters related to food safety [24]. Employing interactions with materials to gather information, HSI offers a nondestructive approach to sample analysis that eliminates the need for complex preprocessing or the addition of chemical reagents, thus avoiding the complications associated with hazardous waste disposal [25]. Capable of performing a single test to assess multiple parameters, HSI is ideally suited for comprehensive evaluations of food safety [26]. Additionally, HSI boasts robust spatial distribution and localization capabilities, which are instrumental in precisely identifying and segregating problematic areas in food products [27]. Area scanning is common in multispectral imaging (MSI), using fewer wavelengths than HSI. As an HSI derivative, MSI acquires narrow-band images at discrete wavelengths. Its primary function is to determine unique wavelength characteristics for each pixel of the observed object, making MSI especially important in the food industry [28].

Raman spectroscopy, as a complementary tool to infrared spectroscopy, has been widely used for food safety detection [29]. However, some samples exhibit strong fluorescence interference that obscures the Raman signals [30]. The limited sensitivity of this technique restricts its practical applications [31]. To overcome these limitations, researchers have made modifications to Raman spectroscopy, leading to the development of SERS [32]. SERS notably boosts molecular Raman signals through electromagnetic fields and chemical effects from metal nanostructures (Fig. 1D). The mechanism involves electromagnetic enhancement, achieved via localized surface plasmon resonance. This occurs when the frequency of incident light approaches the oscillation frequency of free electrons on the metal surface, creating strong localized electromagnetic fields that greatly enhance the Raman scattering intensity of molecules within. Another mechanism is chemical enhancement, occurring when charge transfer effects arise from interactions between the metal substrate and molecules adsorbed on its surface [33]. Studies have shown that SERS can enhance the Raman signals of target molecules by factors of 10^5^ to 10^6^, demonstrating its high sensitivity, specificity, nondestructive nature, and rapid response [34]. These characteristics make SERS a promising tool in food safety detection. For foodborne pathogens, the detection limit can be as low as a few to several tens of CFU/ml. Functionalized nanoparticles are used to separate pathogens from samples. By adding nanoparticles with signal probes, a “sandwich” structure is formed, creating a “hot spot” effect that enhances the Raman signals. The pathogen concentration is quantified by detecting the signal intensity of the reporter molecule [35]. Additionally, Raman reporter molecules possess large Raman scattering cross-sections and narrow characteristic peaks, making them suitable for the simultaneous detection of multiple pathogens [36]. SERS offers ultrahigh sensitivity, enabling the quantitative analysis of trace compounds. Xie et al. [37] utilized a novel flexible SERS substrate made of polydimethylsiloxane film and 3D Au nanostructures for on-site detection of pesticide residues on fruits, achieving a detection limit as low as 9.3 × 10^−9^ M. However, food formulations often contain complex components like proteins, fats, and carbohydrates, which may produce strong Raman signals that interfere with the characteristic peaks of target substances [38]. Machine learning and deep learning algorithms can be employed to automatically calibrate or correct spectral signal variations across different substrate batches, enhancing signal reproducibility and quantitative accuracy. Hajikhani et al. [39] proposed a novel method combining SERS and the transformer model for the rapid detection of pesticide residues in agricultural products. This method utilizes gold–silver core–shell nanoparticles to enhance SERS signals and employs a machine learning model to achieve high-precision qualitative and quantitative analysis. In addition, due to the high sensitivity and specificity of SERS, other spectroscopic techniques can be used for large-scale preliminary screening. Once the target area is located, SERS can be used to accurately confirm and identify trace components [40].

THz is a technique for analyzing substances using electromagnetic radiation in the frequency range of 0.1 to 10 THz, positioned between microwaves and infrared light (Fig. 1E). It exhibits both electronic and photonic properties. The most prevalent method within THz spectroscopy is terahertz time-domain spectroscopy (THz-TDS), a time-resolved technique utilizing femtosecond laser pulses that generate and detect terahertz electric fields, with spectral information being obtained via Fourier transform. The THz-TDS system is composed of a femtosecond laser, a THz emitter, a THz receiver, a time delay control system, and a data acquisition and signal-processing system. Femtosecond laser pulses, divided by a beam splitter into pump and probe beams, are used to generate and detect THz waves [41]. Most chemical compounds exhibit a highly specific absorption spectrum that is frequency-dependent within the THz region. By leveraging this characteristic, chemicals in food can be identified and quantified based on their unique THz fingerprint [42]. THz-TDS is widely applied owing to its excellent coherence, nondestructive nature, and high resolution, making it an effective method for detecting foreign objects in food [43], microbial contamination [44], and toxic compounds [45]. In practical applications, the utility of THz spectroscopy in the food industry is largely limited by strong water absorption, which severely attenuates THz radiation [46]. To overcome this challenge, spectral preprocessing techniques and specialized sample preparation methods may offer viable solutions to enhance measurement accuracy and reliability [47]. THz serves as a powerful tool for detecting moisture content in dry food products, where maintaining low water levels is critical for food safety. However, a key limitation of this method is sample thickness, as measurements become unreliable for samples exceeding 1 mm in thickness. To address this issue, low-frequency terahertz measurements can be employed as an alternative. Additionally, samples may be dried or frozen to reduce interference from liquid water, thereby improving measurement accuracy [45]. Although THz has been demonstrated to facilitate rapid detection of harmful chemical substances in food, its limit of detection (LOD) and accuracy remain relatively lower compared to methods such as immunoassays [48]. Further research is required to enhance the LOD and accuracy of predictive models for food contaminants.

LIBS is an elemental analysis technique based on atomic emission spectroscopy (Fig. 1F). When a high-energy-density pulsed laser is focused on the surface of a sample, the intense power density of the laser rapidly heats a localized area of the sample to extremely high temperatures (over 30,000°C), causing rapid vaporization, melting, and partial ionization to form a transient plasma. As the plasma cools, excited electrons and ions return to lower energy states, emitting characteristic photons that constitute the emission spectrum of the plasma. This spectrum is analyzed to identify the types and concentrations of elements in the sample [49]. LIBS, which requires no sample preparation, offers noncontact measurements, minimal sample damage, and rapid analysis. It is effective not only for detecting heavy metals [50] and pesticide residues [51], but also for identifying food components and assessing authenticity [52], as well as enabling simultaneous multielement detection in complex samples [53], thus providing robust technical support for ensuring food safety. However, detecting trace elements with LIBS is challenging due to its relatively low spectral sensitivity. Common methods to enhance LIBS sensitivity include utilizing dual-pulse laser excitation and employing additional energy sources or tunable lasers to amplify plasma emission, which lowers the detection limits for trace elements [54]. To further enhance the quantitative and elemental mapping capabilities of LIBS, Nanou et al. [55] proposed a LIBS method combined with machine learning (such as principal component analysis [PCA], linear discriminant analysis [LDA], and support vector machine [SVM]) for real-time detection of extra virgin olive oil (EVOO) adulteration with lower-quality oils (pomace, soybean, sunflower, and corn oils). Results demonstrated high efficiency and accuracy, achieving nearly 100% classification and prediction accuracy in distinguishing pure EVOOs from adulterated samples and identifying specific adulterant types. Wen et al. [56] achieved ultrasensitive detection of lead in water by integrating resin enrichment technology with LIBS assisted by laser-induced fluorescence. This approach yielded a remarkable LOD of 88 ng/l, representing a full order of magnitude improvement compared to conventional resin-enriched LIBS alone. In addition, the integration of LIBS with liquid-to-solid phase conversion techniques has been demonstrated to notably enhance spectral signals while effectively reducing detection limits [57].

#### Electrochemical detection technology

Electrochemical detection technology operates based on electrochemical reactions for substance analysis. In a typical setup, a conventional 3-electrode system (working electrode, reference electrode, and counter electrode) is immersed in an electrolyte solution containing the analyte. This configuration facilitates redox reactions that generate measurable current signals. According to the laws of electrolysis established by Faraday, the reaction current exhibits a linear relationship with analyte concentration, enabling quantitative analysis through current measurement. Electrochemical sensors are widely adopted in food analysis due to their inherent advantages, including miniaturization potential, cost-effectiveness, high sensitivity and selectivity, and rapid response. Notably, they achieve high analytical accuracy even in complex food matrices with minimal sample volumes, making them ideal for on-site applications [58]. Recent advancements in this field have diversified detection methodologies to address varying environmental and analytical requirements. Based on differences in analyte physicochemical properties and detection mechanisms, electrochemical detection techniques can be categorized into direct and indirect approaches.

Direct electrochemical detection relies on redox reactions involving inherent electroactive functional groups within the molecular structure of the analyte. However, when using bare electrodes, the detected signals are often considerably weakened due to the poor electrochemical activity of many target molecules. This limitation has led to widespread adoption of electrode surface modifications employing various nanomaterials (e.g., metallic nanoparticles and graphene oxides) [59,60] or biomaterials (e.g., proteins and polymers) [61]. Such modifications primarily function to substantially increase the active surface area of electrode while simultaneously enhancing electron transfer kinetics and introducing additional catalytically active sites, which together can boost detection sensitivity by several orders of magnitude.

For analytes exhibiting inherently low electrochemical activity, the incorporation of redox mediators (e.g., potassium ferricyanide and methylene blue) serves to amplify detectable signals through enhanced electron transfer processes [62]. An alternative approach involves indirect detection methodologies utilizing immobilized biorecognition elements, including enzymes, antibodies, and DNAzymes, on electrode surfaces. In these systems, specific target binding events induce quantifiable modifications in the electrochemical behavior of reporter molecules. This principle has been implemented through various sensor architectures, notably electrochemical enzymatic biosensors employing glucose oxidase for detection [63], functional nucleic acid-based sensors incorporating aptamer recognition systems [64], and molecularly imprinted polymer sensors that mimic antibody–antigen interactions [65].

**Fig. 2.**
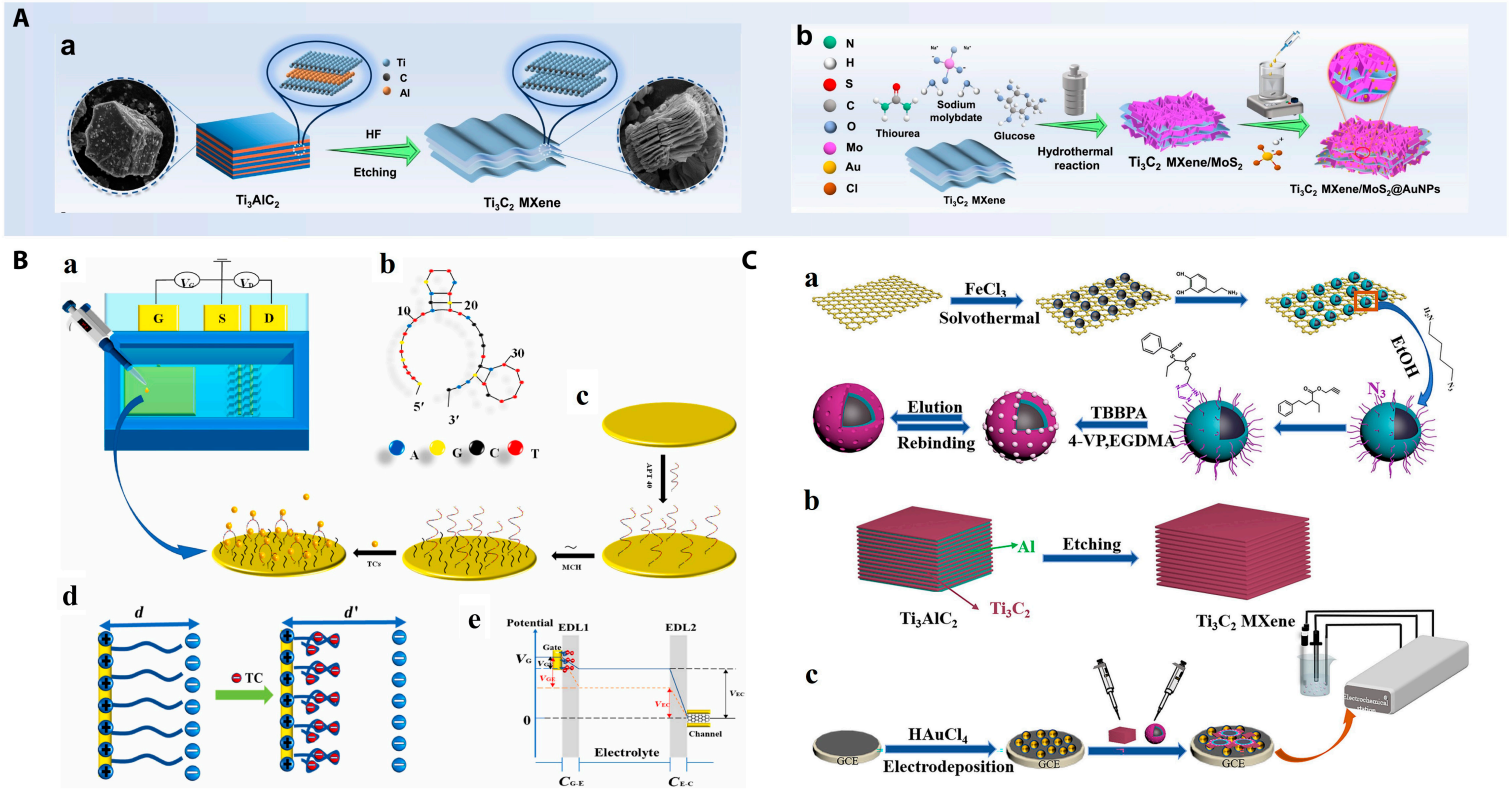
(A) (a) Preparation process of Ti_3_C_2_ MXene nanosheets and synthesis of Ti_3_C_2_ MXene/MoS_2_ and (b) Ti_3_C_2_ MXene/MoS_2_@AuNPs nanocomposites. Reproduced with permission [66]. Copyright 2025, Elsevier. (B) Schematic diagrams illustrate (a) the miniaturized Apt-SGGT biosensor structure, (b) the 2D structure of APT40, (c) the modification process using APT40 and the detection of TC by immobilized APT40 probes, (d) alterations in the bilayer before and after the addition of TC, and (e) charge recombination at the electrode surface triggers potential fluctuations in the double electric layer. Reproduced with permission [67]. Copyright 2024, Elsevier. (C) (a) Preparation procedure of GO@Fe_3_O_4_@MIP, (b) MXene, and (c) TBBPA-imprinted electrochemical sensor. Reproduced with permission [69]. Copyright 2022, Springer Nature.

Electrochemical enzyme sensors employ enzymes as recognition elements to catalyze substrate-specific reactions, generating electroactive species that modulate the electrochemical response. As illustrated in Fig. 2A, Zhang et al. [66] developed an acetylcholinesterase-based biosensor using a Ti₃C₂ MXene/MoS₂@AuNPs nanocomposite for organophosphorus pesticide detection in fruits. This sensor demonstrated remarkable sensitivity, enhanced conductivity, and operational stability, proving its efficacy in complex food matrices. Functional nucleic acids (e.g., aptamers and DNAzymes) enable target recognition through high-affinity binding or catalytic activity. In electrochemical aptamer sensors, immobilized aptamers undergo conformational changes upon target binding, perturbing the interfacial electron transfer. For instance, Dou et al. [67] designed a miniaturized tetracycline biosensor by functionalizing a graphene field-effect transistor with aptamers (Fig. 2B). The sensor utilized 2 complementary detection methods: transfer characteristic curve analysis and chronoamperometric measurement, achieving respective detection limits of 2.073 and 100 pM in validated skim milk samples. The biosensor proved effective for skim milk analysis. Similarly, Zhao et al. [68] engineered a Pb^2+^ biosensor using a solution-gated graphene transistor (SGGT) modified with a Pb^2+^-specific DNAzyme. Unlike conventional DNAzyme probes, this design leveraged a noncleavable binding mode, achieving an ultralow detection limit (0.39 μg/l) in milk samples, underscoring its potential for on-site monitoring. Molecular imprinting technology facilitates the creation of polymer matrices containing 3-dimensional recognition cavities with tailored molecular geometry and functional group alignment, achieving high specificity for target molecule binding. The extensive utilization of tetrabromobisphenol A (TBBPA) in industrial manufacturing has led to its persistent accumulation in aquatic environments, raising notable concerns due to its endocrine-disrupting effects and ecological toxicity. As illustrated in Fig. 2C, Shao et al. [69] addressed this analytical challenge by developing an electrochemical sensor through sequential modification of a glassy carbon electrode with MXene nanosheets and gold nanoparticles, followed by drop-coating of molecularly imprinted polymers. This architecture demonstrated exceptional selectivity for TBBPA detection in aqueous systems, with validation studies reporting recovery rates of 97.1%–106% during real-water sample analysis.

#### Machine olfaction technology

**Fig. 3.**
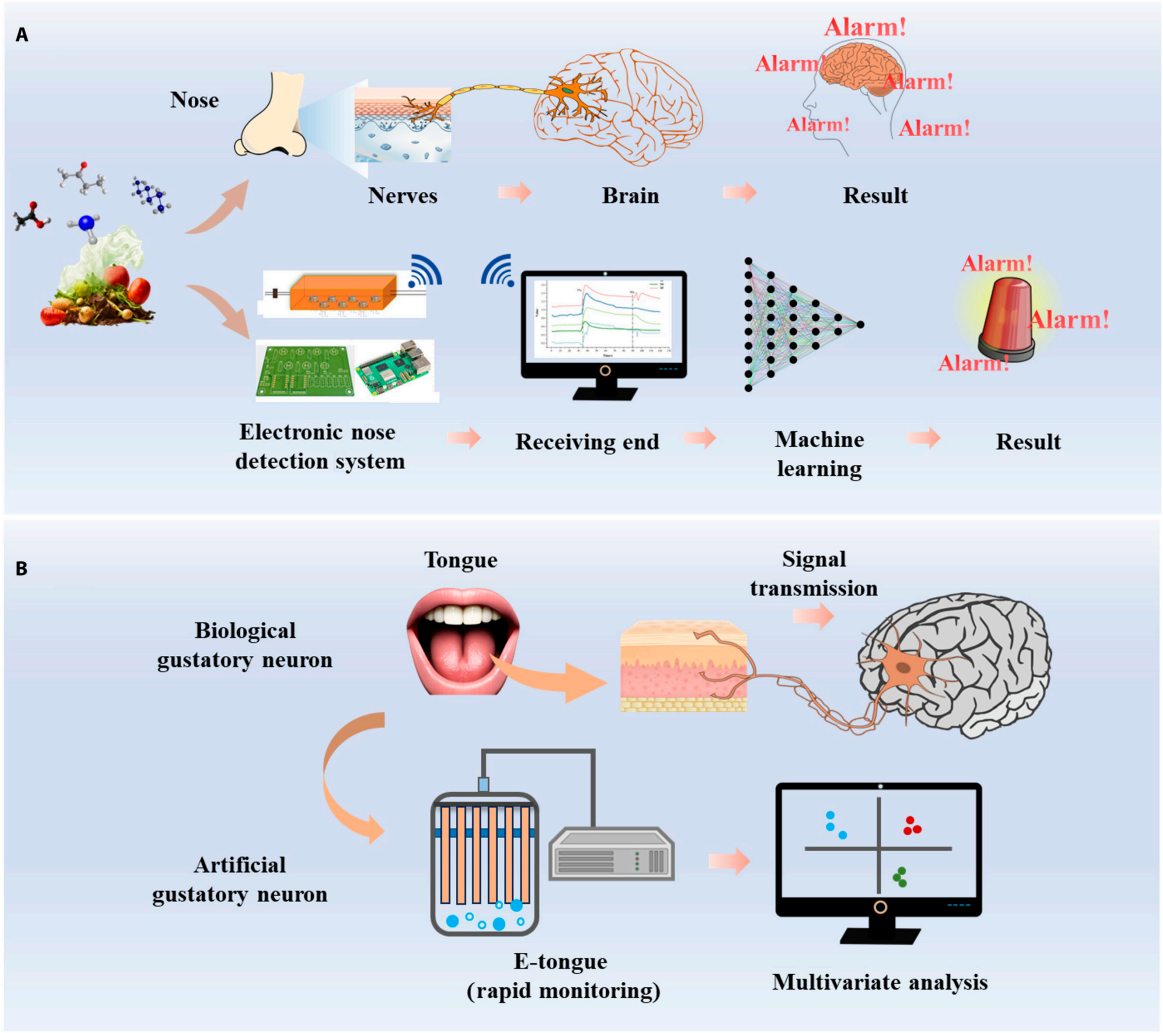
(A) E-nose: mimics human smell using sensors to detect odors, processed by machine learning for alert-based results. (B) E-tongue: imitates human taste, capturing flavor signals analyzed by computers for rapid response.

Machine olfaction technology, also referred to as artificial olfactory system or electronic nose (E-nose), simulates human olfaction (Fig. 3A). It detects and identifies volatile compounds using sensors and utilizes data processing algorithms to analyze their chemical composition and concentration. In the 1980s, Persaud and Dodd [70] integrated array sensors with pattern recognition algorithms to develop a device capable of identifying 21 distinct odors, marking the inception of the E-nose. The key advantages of E-nose technology include reduced sample volume needs and high-speed analysis capabilities. However, they face challenges in identifying or quantifying individual compounds present in food [71].

E-nose primarily comprises 3 components: a gas sensor array, a signal-processing unit, and pattern recognition algorithms. The gas sensor array, as the core of the system, detects volatile organic compounds (VOCs) and converts chemical signals into electrical signals, directly determining the performance of the system. Common gas sensors include metal oxide semiconductor (MOS) sensors, quartz crystal microbalance sensors, conductive polymer sensors, and surface acoustic wave sensors [72]. The signal-processing unit amplifies, filters, and digitizes analog signals, while pattern recognition algorithms, such as ANN, SVM, and fuzzy logic, classify the processed data to achieve odor identification [73]. E-nose has emerged as an effective technology for detecting VOCs, offering a rapid method for identifying food contamination, adulteration, and origin authentication, which is crucial for consumer safety and cost-effective solutions. However, E-nose generates high-dimensional time-series raw signals for target gases, which can be noisy, redundant, and difficult to interpret. To address this challenge, advanced E-nose systems are integrated with machine learning algorithms to enable accurate identification of gas molecules [74]. Makarichian et al. [75] integrated E-nose technology with SVM, LDA, and backpropagation neural network (BPNN) methods to rapidly and accurately predict fungal contamination in garlic samples. Employing a 9-sensor MOS array and selecting the decay severity index as an auxiliary parameter, their study demonstrated that the classification accuracy of BPNN improved progressively with increasing infection duration. By the eighth day of infection, the BPNN algorithm successfully discriminated between samples at different contamination levels. Leggieri et al. [76] developed a combined E-nose and ANN system for rapid detection of aflatoxin B_1_ (AFB_1_) and fumonisins in corn samples. Analyzing multiple production batches using a portable E-nose with 10 MOS sensors, the integrated ANN model demonstrated 78% accuracy for AFB_1_ detection and 77% for fumonisins, confirming the effectiveness of this system in identifying mycotoxins within regulatory limits.

#### Machine gustatory technology

Machine gustatory technology is a technique that utilizes sensor technology and advanced algorithms to emulate the taste perception abilities of humans or animals, with the electronic tongue (E-tongue) system representing the most typical machine taste system (Fig. 3B). E-tongue technology, which leverages biomimetic principles to mimic the taste functions of the human tongue, operates by initiating interactions (such as charge transfer, molecular adsorption, or desorption) between the molecules in a test sample and the sensors of E-tongue, producing a series of electrical signals. These signals are captured and converted into mathematical signals for data processing and analysis, thereby identifying and differentiating various taste types and quantitatively assessing their intensity and complexity [73]. Taste sensors serve as the core components of the E-tongue system. These sensors differ in type and principle, such as potentiometric, voltametric, and optical taste sensors. Through their different mechanisms, these sensors detect gustatory components in food and generate corresponding signals. These signals are then analyzed to conduct both qualitative and quantitative assessments, with common multivariate analysis methods such as PCA utilized to process the multidimensional data from the E-tongue. The simplicity of operation and low cost render E-tongue particularly suitable for real-world applications [77]. Cheng et al. [78] introduced a voltammetric E-tongue technology based on the “reference sample comparison method” to evaluate the shelf life of fresh milk. By introducing reference samples, the method quantifies the quality difference between fresh milk samples with varying shelf lives and the reference samples. This approach, combined with a first-order kinetic model and the Arrhenius equation, successfully established a shelf life prediction model for fresh milk stored at 4 °C. The results demonstrated that this method exhibited superior predictive performance in assessing the shelf life of overall quality, microbial safety, and sensory quality, markedly outperforming the traditional “many-to-one” model. Specifically, it improved prediction accuracy by 11.14% to 17.17% for overall quality shelf life evaluation and by 14.86% to 44.47% for other metrics. In addition to the voltammetric E-tongue, another prevalent type is the potentiometric E-tongue. The potentiometric E-tongue relies on polymer membrane ion-selective electrodes and ion-selective field-effect transistors, detecting analytes by measuring potential differences under zero-current conditions [79]. Gil et al. [80] designed a custom-made E-tongue consisting of 6 electrodes (Au, Ag, Cu, Pb, Zn, and C) and a reference electrode. This device was used to assess changes in physicochemical, microbial, and biochemical parameters in refrigerated pork loin during storage. The responses from the E-tongue showed relatively good correlations with certain degradation indices, such as pH, microbial counts, and nucleotide concentrations.

#### Others

In addition to the aforementioned detection technologies, acoustic and magnetic methods have been considered in preliminary application research for food safety detection. Ultrasonic waves, with frequencies above 20 kHz, demonstrate excellent propagation characteristics owing to their high frequency and experience minimal loss when traveling through solids and liquids [81]. Ultrasonic testing typically employs the pulse-echo method, where pulses of ultrasonic waves are emitted and then reflected from the surface of an object, are captured by a receiver, and are converted into electrical signals for analysis [82]. In food testing, ultrasonic devices usually immerse the probe in the liquid food or attach it to the container walls, operating at a certain distance on both sides of the food. Depending on the measurement mode, 2 probes can transmit and receive ultrasonic waves either once or multiple times, recording the interval between transmission and reception and other related data to assess whether the food quality level meets the standard requirements, and further determine the potential degree of spoilage [83].

Nuclear magnetic resonance (NMR) technology is an analytical method based on the spinning behavior of atomic nuclei in a magnetic field. Its core mechanism is the resonance of magnetically active nuclei under an applied external magnetic field and radiofrequency radiation, enabling detection through spin-state transitions. By measuring the NMR signals in a sample, information about molecular structure, composition, and interactions can be obtained [84]. In practical applications, NMR technology can induce nuclear spin oscillations at the Larmor frequency by applying appropriate radiofrequency pulses to generate energy-absorption signals. Subsequently, the nuclei undergo 2 different relaxation processes: longitudinal relaxation (T_1_), which results in energy transfer from the excited state to the surrounding spinning nuclei, and transverse relaxation (T_2_), which involves energy transfer between nuclei, leading to a loss of coherence [85]. The intensity of the NMR signal is proportional to the number of excited nuclei. Time-domain data are transformed into frequency-domain data through Fourier transform, producing a typical NMR spectrum. Each signal in the spectrum exhibits a specific chemical shift value that reflects the chemical environment of the surrounding nuclei [86]. NMR technology boasts several advantages due to its nondestructive, high-resolution, multidimensional, and nonspecific nature, making it highly effective in the detection of additives, contaminants, residues, and authenticity issues in food.

### Signal-processing technologies in intelligent sensing

Intelligent sensing technology accurately captures physical and chemical information from food samples with high precision, providing strong data support for food safety detection. The signal-processing technologies upon which these systems rely are crucial for interpreting and utilizing this information; their complexity and importance cannot be overlooked. Effective signal processing not only dramatically enhances the quality of the detection data but also prepares it for subsequent analysis by removing noise, correcting baselines, normalizing, and executing other optimization steps.

Preprocessing steps are a critical component in enhancing the accuracy of food safety detection, with different technologies adopting various methods tailored to their specific needs. In spectroscopic techniques, common preprocessing steps include noise removal, baseline correction, and signal normalization, which improve the accuracy and comparability of subsequent data analyses [87]. Common NIRS often requires multiple mathematical transformations, such as logarithmic transformations [88], to enhance small absorption differences, whereas HSI may need spatial filtering to remove background noise [89]. For E-nose and E-tongue systems, preprocessing steps such as signal smoothing, baseline drift correction, compression, and response normalization are typically required to reduce response differences between sensors [90]. For acoustic signals, certain techniques such as sound amplification and high-pass filtering are commonly used to eliminate low-frequency environmental noise and accurately capture the required acoustic signals [91].

Feature extraction is a crucial step in data analysis because it improves data quality, reduces dimensionality, and enhances interpretability. For spectral data, feature extraction tools are commonly used to select optimal feature wavelengths from spectral datasets, thereby removing redundant information. Popular techniques are sequential projection algorithm (SPA), competitive adaptive reweighting sampling (CARS), uninformative variable elimination (UVE), and interval partial least squares (iPLS) [92]. Feature extraction techniques for E-nose and E-tongue can be categorized into 3 types: the first involves fitting functions, the second extracts features directly from the raw response functions of sensors, and the third involves transformations using mathematical functions [73]. In addition, widely used dimensionality reduction techniques such as PCA and independent component analysis are key tools for reducing dimensions. For univariate data, certain techniques such as wavelet transform, kernel PCA, and LDA are often employed for dimension reduction and feature extraction [93]. In electrochemical detection, it is common to extract the peak features that identify the redox properties and concentrations of chemicals. In electrochemical impedance spectroscopy, resistance and impedance changes can reveal the electrochemical characteristics of samples [94]. When using acoustic and magnetic technologies for detection, attention is typically given to changes in the frequency, amplitude, and phase of sound or magnetic waves [95,96]. Overall, these feature extraction techniques not only accurately capture the key attributes of food but also provide robust data support for subsequent safety monitoring.

### Modeling technologies in intelligent sensing

#### Machine learning

**Fig. 4.**
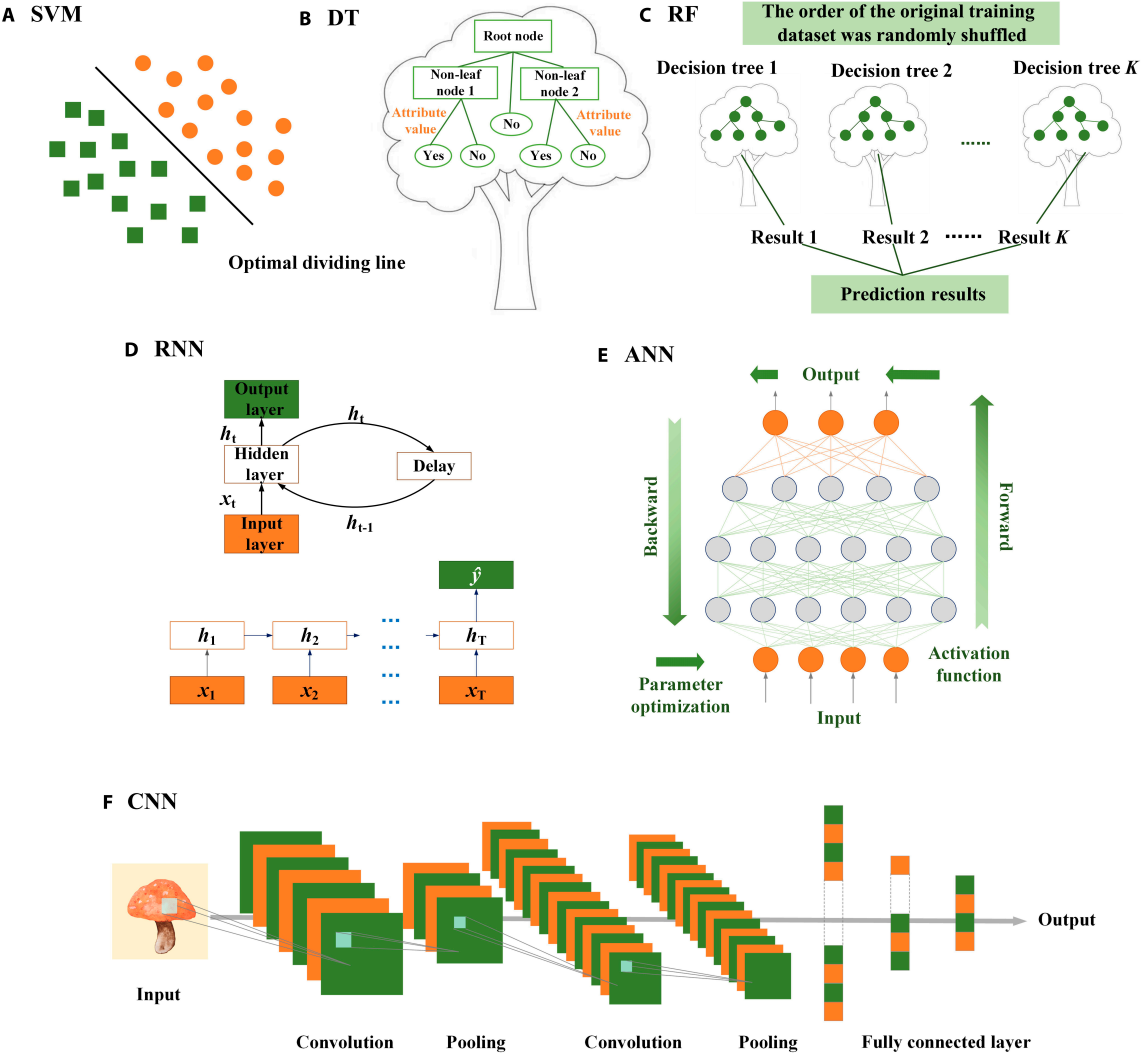
Machine learning algorithm models: (A) Support vector machine (SVM): finds the optimal dividing line or hyperplane that best separates different classes of data. (B) Decision tree (DT): uses a tree-like model of decisions and their possible consequences to classify data. (C) Random forest (RF): constructs a multitude of decision trees at training time and outputs the class that is the mode of the classes of the individual trees. (D) Recurrent neural network (RNN): processes sequences by maintaining a state that represents information calculated from previous inputs. (E) Artificial neural network (ANN): consists of layers of interconnected nodes or neurons that process input data through weighted connections for various types of classification and prediction tasks. (F) Convolutional neural network (CNN): employs a hierarchy of layer-wise convolutions specifically designed to process pixel data, ideal for image recognition tasks.

The selection of algorithms and models is pivotal for achieving efficient intelligent sensing, requiring alignment with data characteristics and specific objectives. Linear regression models, known for their simplicity and effectiveness in linear scenarios, have been widely adopted in rapid food safety detection to calculate safety parameters. Their results are consistent with traditional methods while offering superior efficiency. However, real-world data often involve complex nonlinear relationships, prompting the use of traditional machine learning models such as SVM, decision trees (DT), and random forest (RF) for low-dimensional data analysis (Fig. 4A to C) [97]. For instance, Xie et al. [98] integrated NIRS with RF and SVM to predict carcinogen levels in coffee, where RF outperformed SVM with calibration (*R*^2^ = 0.98) and prediction (*R*^2^ = 0.92) accuracies. Similarly, Wu et al. [99] leveraged DT models on agricultural data for early disease detection in crops. Despite their success in low-dimensional contexts, these models face limitations in processing high-dimensional data (e.g., images or audio). Such challenges necessitated a fundamental shift toward deep learning, which leverages automated feature extraction to handle complex sensing tasks.

#### Deep learning

The deep learning revolution originated from the seminal work of Hinton et al. [100], who, in 2006, proposed deep belief networks (DBNs) alongside efficient training algorithms. This breakthrough demonstrated the potential of hierarchical feature learning, paving the way for architectures tailored to high-dimensional data. Among these, convolutional neural networks (CNNs, Fig. 4F) and recurrent neural networks (RNNs, Fig. 4D) emerged as dominant paradigms, addressing the limitations of traditional models in image and sequence processing, respectively [101].

CNNs are composed of distinct layers. The input layer serves as the initial layer, receiving data such as images of food. The output layer produces the final prediction, such as the freshness of the food. The hidden layers, positioned between the input and output layers, comprise convolutional layers, pooling layers, and fully connected layers. These layers often incorporate rectified linear unit (ReLU) activation functions to enhance the expressive capacity of the model [102]. Wang et al. [103] applied the CNN algorithm to a colorimetric microneedle sensor (CMS) for visual monitoring of meat freshness. The CMS changes color in response to pH variations caused by meat spoilage. A total of 2,921 images of CMS affixed to meat with different freshness were imported into the CNN model as the training source. Through convolutional processing of the CMS color features, the model classified meat freshness into 3 categories: “fresh”, “less fresh”, and “spoiled”, achieving an accuracy of approximately 95.3%. The application of CNN enabled rapid and precise identification of meat freshness, substantially improving the efficiency and accuracy of meat quality assessment compared to traditional manual inspection methods.

While CNNs excel at extracting spatial features for static image classification tasks like meat freshness detection, they are less suited for analyzing data with temporal dependencies or dynamic changes over time. For such scenarios, researchers often turn to RNNs. However, traditional RNNs face limitations such as vanishing or exploding gradients, which hinder their ability to learn long-term dependencies in sequences. To address these challenges, enhanced architectures like long short-term memory network (LSTM) [104] and gated recurrent unit [105] were introduced. These variants employ gating mechanisms to selectively retain or discard information over time, enabling RNNs to capture critical temporal patterns while mitigating gradient-related issues. This capability makes them particularly suited for tasks like continuous food safety monitoring, production process analysis, or risk prediction, where understanding temporal trends is critical [106]. RNN is very important in identifying the authenticity of food products and is mainly used to analyze spectral data. Wu et al. [107] synthesized a highly sensitive star-shaped SERS substrate to rapidly detect adulteration of EVOO with 2 phthalate esters. By leveraging LSTM networks to process spectral data in the 1,000 to 1,800 cm^−1^ range, they achieved exceptional performance: After optimizing the data using t-distributed stochastic neighbor embedding (t-SNE) for dimensionality reduction, the LSTM model demonstrated 98.15% accuracy in identifying adulterated olive oil, notably outperforming conventional full-spectrum analysis methods. Beyond this, LSTM has also been applied to assess foodborne hazards such as aflatoxins, leveraging spectral reconstruction and model parameter compression techniques to address data imbalance and reduce computational complexity [108].

#### Semi-supervised learning and unsupervised learning

While supervised learning frameworks have demonstrated remarkable success in food safety tasks, their reliance on large annotated datasets poses challenges in real-world scenarios where labeled data may be scarce or costly to acquire. To address these limitations, researchers are increasingly adopting semi-supervised learning and unsupervised learning paradigms. The core idea of semi-supervised learning is to improve the model’s generalization ability by utilizing the underlying structural information in unlabeled data. In contrast to food safety assessments, which is often time-consuming and expensive, semi-supervised learning offers substantial advantages, as it requires experts to evaluate and label the data. Common semi-supervised learning approaches are self-training, co-training, graph-based semi-supervised learning, and generative adversarial networks (GANs). Recent studies have introduced semi-supervised learning models for pseudo-label monitoring [109] and multilabel food recognition [110], which further improve the performance of semi-supervised learning in food detection.

In unsupervised learning, the training data are unlabeled. Clustering and dimensionality reduction are 2 typical applications of unsupervised learning. PCA is a key technique for reducing data dimensionality, achieved by projecting high-dimensional data onto orthogonal directions (principal components) with the maximum variance, thereby preserving critical information while reducing dimensions [111]. PCA can rapidly distinguish EVOO from other edible oils based on triglyceride profiles. Similarly, K-Means clustering has demonstrated excellent performance in automated quality and safety grading. Green et al. [112] employed clustering methods to standardize the freshness assessment of 7 fish species, comparing K-Means, K-Means++, AHC-S, AHC-W, DBSCAN, and spectral clustering, with K-Means and K-Means++ showing superior performance. These cases illustrate that unsupervised learning can uncover actionable patterns from unlabeled food data by optimizing intra-class compactness and inter-class separability, making it particularly valuable for exploratory research in dynamic food safety studies where prior knowledge is limited.

#### Model evaluation

Model evaluation metrics are essential tools for assessing the performance of predictive models. Different types of tasks (e.g., classification and regression) generally require distinct evaluation metrics. For classification problems, metrics such as accuracy, precision, recall, and the F_1_ score are commonly used to assess model performance. In contrast, for regression tasks, metrics like mean squared error (MSE), root mean squared error (RMSE), mean absolute error (MAE), and the coefficient of determination (*R*^2^) are typically employed to evaluate the effectiveness of model. Lower values of MSE, RMSE, and MAE indicate better model fit, while an *R*^2^ value closer to 1 signifies a stronger explanatory power [113]. Detailed formulas for these metrics are available in the referenced literature [114,115].

$$\text{Accuracy}=\frac{\mathrm{TP}+\mathrm{TN}}{\mathrm{TP}+\mathrm{TN}+\mathrm{FP}+\mathrm{FN}}$$ (1)

$$\text{Precision}=\frac{\mathrm{TP}}{\mathrm{TP}+\mathrm{FP}}$$ (2)

$$\text{Recall}=\frac{\mathrm{TP}}{\mathrm{TP}+\mathrm{FN}}$$ (3)

$$F1\;\text{core}=2\cdot \frac{\text{Precision}\cdot \text{Recall}}{\text{Precision}+\text{Recall}}$$ (4)

$$\mathrm{MSE}=\frac{1}{n}\sum_{i=1}^{n}{\left(y_{i}-{\hat{y}}_{i}\right)}^{2}$$ (5)

$$\text{RMSE}=\sqrt{\frac{1}{n}\sum_{i=1}^{n}{\left(y_{i}-{\hat{y}}_{i}\right)}^{2}}$$ (6)

$$\mathrm{MAE}=\frac{1}{n}\sum \left|y_{i}-{\hat{y}}_{i}\right|$$ (7)

$$R^{2}=1-\frac{\sum_{i=1}^{n}{\left(y_{i}-{\hat{y}}_{i}\right)}^{2}}{\sum_{i=1}^{n}{\left(y_{i}-\bar{y}\right)}^{2}}$$ (8)

TP (true positive): the number of samples that the model correctly predicts as positive classes.

TN (true negative): the number of samples correctly predicted by the model to be in the negative category.

FP (false positive): the number of samples that the model incorrectly predicts as positive.

FN (false negative): the number of samples that the model incorrectly predicts as negative.

$y_{i}$: actual value of sample *i*; ${\hat{y}}_{i}$: predicted value for sample *i*; $\bar{y}$: average of actual values.

*n*: total sample size.

## The Application of Intelligent Sensing Technology in Food Safety Detection

### Microbial hazard detection

Foodborne diseases have emerged as a major public health concern, with pathogenic bacteria and molds being the primary culprits [116]. Pathogens such as *Salmonella* and *Shigella* can cause food poisoning and illness, whereas certain serotypes of *Escherichia coli* are highly pathogenic, leading to diarrhea and other extraintestinal infections. Toxins produced by molds, such as aflatoxin, can cause cancer if ingested over a long period. Given these potential threats, selecting more efficient and accurate detection methods to ensure food safety is crucial. Intelligent sensing technologies provide unique advantages in the detection of microbial hazards. They offer rapid, efficient, accurate, reliable, and fully automatic monitoring in real time, making them essential tools for ensuring food safety. Table 1 presents specific examples of intelligent sensing technology applications in the detection of food microbial hazards.

**Table 1.** Detection of intelligent sensing techniques in food microbiological hazards

Type	Food product	Safety attribute	Method	Methodological application	Performance	Reference
Viruses	Cauliflower	Bacterial soft rot	MV	A modified YOLOv8 model called Cauli-Det	Classification accuracy 93.2%, recall 82.6%, mean average precision 91.1%	[150]
	*Salmo salar*	Bacterial numbers	NIR (800–2,500 nm)	PLS	Calibration equation: *R*^2^ = 0.95 and RMSE = 0.12 log CFU/g	[151]
					Validation curve: *R*^2^ = 0.64 and RMSE = 0.32 log CFU/g	
	Kombucha	Bacterial concentration	Vis-NIR	BP-ANN, ELM-ANN, RBF-ANN	RBF-ANN best (RPD = 6.7878), BP-ANN with a max error of 0.0169 Au	[152]
	*Longissimus dorsi* muscle of beef	Microbial load	HSI	PLS-R and data fusion	4 °C: RMSE_P_ 0.58 log CFU/g, RPD_p_ 4.13, $R_{p}^{2}$ 0.96	[153]
					10 °C: RMSE_P_ 0.97 log CFU/g, RPD_P_ 3.28, $R_{p}^{2}$ 0.94	
	Pork and milk	*Salmonella typhimurium*	Electrochemical sensor	A novel molecularly imprinted polymer sensor based on screen-printed electrodes	Detection limit approximately of 10^1^ CFU/ml and a detection time of only 4 min	[154]
	Pork	*Salmonella typhimurium*	E-nose	SVMR, GA, PSO, and GS	Predictive accuracy: GA-SVMR > PSO-SVMR > PSO-SVMR > GS-SVMR > SVMR. GA-SVMR ($R_{p}^{2}$ = 0.989; RMSE_P_ = 0.137; RPD = 14.93	[155]
	Milk	*Escherichia coli*, *Klebsiella pneumoniae*, and *Salmonella enterica*	E-tongue	Data processing: PCA and LDA	Classification accuracy 92.5%, the E-tongue with gold electrodes accurately distinguished decreasing *E. coli* concentrations (1 × 10^6^ to 1 × 10^−2^ CFU/ml) with 98.7% success.	[156]
				Data classification: SVM and k-NN		
Mold	Unhulled paddy	*Aspergillus nidulans*, *Aspergillus niger*, *Penicillum citrinum*, *Aspergillus oryzae*, and *Aspergillus versicolor*	MV	SVM, BPNN, CNN, and DBN	The DBN model excels in mold species identification, while the CNN model is fast and accurate for mold colony region detection.	[157]
	Peanut	*Aspergillus* spp.	NIR and E-nose	Classification: LDA	Classification accuracy: NIR 92.11%, E-nose 86.84%	[158]
				Quantification: PLSR	Quantification: NIR ($R_{p}^{2}$ = 0.886, RPD = 3.0, LOD = 0.578 log CFU/g), E-nose ($R_{p}^{2}$ = 0.785, RPD = 2.1, LOD = 0.808 log CFU/g)	
	Green tea	Total mold colony count	HSI (431–962 nm)	CARS, SVR, GA, PSO	CARS-GA-PSO-SVR achieved accuracy with an $R_{p}^{2}$ of 0.9577 and an RMSE of prediction set of 0.1140 lg (CFU/g)	[159]
	Pumpkin seeds	Degree of mold	THz-TDS	SVM, RF, CNN	Classification accuracy: CNN best 96%	[160]
	Spanish-style green olives	Spoilage mold species	E-nose	PCA and PLS-DA	PC_1_ and PC_2_ explained 70.23% and 23.27% of the total variance, classification accuracy 95.5%	[161]
	Corn kernels	Mildew-damaged kernels	Impact acoustics	Pretreatment: EEMD	Classification accuracy 99.3%	[162]
				Classification: PSO-SVM		

MV, machine vision; NIR, near-infrared; PLS, partial least squares; RMSE, root mean squared error; BP-ANN, backpropagation ANN; ELM-ANN, extreme learning machine ANN; RBF-ANN, radial basis function ANN; RPD, relative predictive deviation; HSI, hyperspectral imaging; SVMR, support vector machine regression; GA, genetic algorithm; PSO, particle swarm optimization; GS, grid searching; PCA, principal component analysis; LDA, linear discriminant analysis; k-NN, k-nearest neighbors; BPNN, backpropagation neural network; CNN, convolutional neural network; DBN, deep belief networks; PLSR, partial least squares regression; CARS, competitive adaptive reweighting sampling; SVR, support vector regression; THz-TDS, terahertz time-domain spectroscopy; SVM, support vector machine; RF, random forest; PLS-DA, partial least squares discriminant analysis; EEMD, ensemble empirical mode decomposition

### Chemical hazard detection

Chemical hazards, such as pesticide residues, veterinary drug residues, misuse of food additives, heavy metal contamination, and mycotoxins, can enter the food chain either directly during production and processing or indirectly through environmental pathways. Pesticides are among the primary sources of chemical hazards, comprising insecticides, herbicides, fungicides, and rodenticides [117]. These are widely used in agricultural production to protect crops from pests and other threats. However, pesticides often spread to nontarget species, the air, and water bodies, causing environmental pollution and entering the human body through the food chain. Food additives are substances added to food to enhance its quality, color, aroma, and taste or use for preservation and processing purposes. These additives can be either synthetically produced or derived from natural sources [118]. Although approved food additives are generally considered safe, regulatory limits vary by country, and some manufacturers illegally add harmful substances to increase profits, violating laws and potentially posing serious health risks to consumers. Heavy metal contamination in food is a global safety concern, originating from natural and anthropogenic sources, such as industrial wastewater, emissions, and waste. Heavy metals, which are not easily degraded in the environment, enter the human body via the food chain, leading to chronic poisoning and severe health risks [119]. Moreover, mycotoxins are metabolic products produced by fungi growing in food or feed, which consist of highly toxic and carcinogenic substances (e.g., aflatoxins, ochratoxin A, vomitoxin, and zearalenone), all of which pose serious threats to human and animal health [120]. Table 2 lists the applications of intelligent sensing technology in food chemical hazard detection.

**Table 2.** Detection of intelligent sensing techniques in food chemical hazards

Type	Food product	Safety attribute	Method	Methodological application	Performance	Reference
Pesticide	Jujube	Chlorpyrifos and imidacloprid	HSI (900–1,700 nm), GC-MS	PLS-DA and LWPLSR	E_S_-AWLS-GSD-RC-LWPLSR model yielded *R*_CV_ of 0.757 and 0.898 for chlorpyrifos and imidacloprid	[163]
	Bean, apple, and vegetable	Triazole pesticides	SERS	Au decahedral substrate with high E-field intensity	Low LOD and wide detection range for in situ and simultaneous detection of triazoles in fruit and vegetable samples	[164]
	Cabbage	Acetamiprid (AD) and malathion (ML)	Electrochemical aptasensor	Substrates: PR and NFs/HP-UiO66-NH_2_	Linear range: 10 pM to 0.1 μM	[165]
				Signal amplification: redox molecule/MOF composites	Detection limits: AD (4.8 pM), ML (0.51 pM)	
				Build signal labels: FcCysAu/CeMOF (III, IV) and MB/MOF235	Higher accuracy than HPLC-MS for cabbage samples	
	Apple	Cypermethrin and chlorpyrifos	E-nose	PCA, LDA, SVM	PCA shows the best classification and differentiation ability	[166]
Food additive	Sausages	Sodium nitrite	NIR-HSI	PLSR, PLS-DA	PLSR: *R*_P_ 0.92, RMSEP 15.603 mg/kg	[167]
					Classification accuracy: PLS-DA 91.3%	
	Beverage	Lycopene	SERS	A 2D composite semiconductor, formed by growing ZIF-67 film on BP nanosheets, shows enhanced performance due to efficient charge transfer in the BP/ZIF-67 system	Recovery rates: 86.9% to 93.3%	[168]
	Infant formula	Vanillin	THz-TDS	Enhanced detection sensitivity using quasi-bound states in the continuum, quasi-BIC property	Vanillin has an absorption peak at 1.83 THz	[169]
	Fruit juice	Benzoic acid and chitosan	E-nose	RF, ELM, SVM, PLSR	ELM and RF have higher $R_{s}^{2}$ and lower RMSEs	[170]
	Dessert	Vanillin	Electrochemical sensor	Co_2_NiO_4_@PtCu-MIG as an artificial antibody to construct electrochemical sensors for the selective detection of vanillin	Wide linear range: 0.05–500 μM	[171]
					LOD 3.45 nM	
	Bread and bakery products	Propionic and sorbic acid	^1^H NMR	Short time extraction of sorbic and propionic acid from bread by automatic steam distillation	NMR is fast and reproducible in the quantitative analysis of 2 substances	[172]
Veterinary	Mutton	Pefloxacin	HSI and Vis–NIR	PLSR and data fusion	Low-level fusion: $R_{p}^{2}$ = 0.907 and RMSEP = 0.462	[134]
					Intermediate-level fusion: $R_{p}^{2}$ = 0.940 and RMSEP = 0.375	
	Milk and fish	Quantitative, accurate and multiple	SERS	SERS activity of plasmonic gold nanobipyramid@Silver nanorod (Au NBP@Ag NR) -CsPbX_3_ thin films	Enhancement of Au NBPs@Ag NRs films is 3.6	[173]
	Water and fish	Malachite green	Electrochemical sensor	A dual-mode colorimetric and electrochemical method based on P-CeO_2_NR@Mxene and m-TDN was developed.	Colorimetric mode: 95.4 pM	[174]
					Electrochemical mode: 83.6 fM	
					Colorimetric recovery: 97%–104%, RSD 1.74%–3.62%	
					Electrochemical recovery: 95%–103.7%, RSD 2.03%–4.13%	
Heavy metal	Mussel	Zn, Pb, Cd, and Cu	NIRS (900–1,700 nm)	CDELM	Classification accuracy: Zn 97.53%, Pb 95.67%, Cd 99.00%, Cu 98.8%	[175]
	Water	Cu and Fe	HSI	Feature recognition: RF	Cu: *R*^2^ 0.75, RMSE 0.004, MRE 0.382	[176]
				Predictive model: GA-PLSR	Fe: *R*^2^ 0.73, RMSE 0.036, MRE 0.464	
	Tea	Hg	SERS	Novel dual-mode paper sensor constructed	LOD 0.48 pM	[177]
				AuNPs as colorimetric signal molecules and SERS substrates	Increased sensitivity: 200-fold for colorimetry, 500-fold for SERS	
	Water	Pb and Ni	THz	Microalgae as a medium	Optimal time for testing: Pb^2+^ 6 h, Ni^2+^ 18 h	[178]
				PLS and PCA	Accuracy: Pb^2+^ 100%, Ni^2+^ 93.2%	
					Detection accuracy: increase from 10 to 1 ng/ml	
	Tea infusion	Cr and Cu	LIBS combined with electrostatic spinning	Modification of electrostatically spun nanofibrous membranes by AuNPs and AgNPs	LOD: Cr 5 μg/l and Cu 10 μg/l	[179]
					Recovery rate: Cr 99%–106%, Cu 99%–108%	
	Water	Pb	E-tongue	Evaluation of ternary nanocomposites based on electrospun nanofibers, cellulose nanowhiskers ,and silver nanoparticles as a sensing layer for the electrical detection of heavy metals	Effectively differentiates between pure water and aqueous solutions contaminated with Pb^2+^ at concentrations as low as 10 nmol l^−1^.	[180]
	Milk	Cd, Pb, Cu, and Hg	Electrochemical sensor	Heavy metal ions mixed with Fe_3_O_4_@SiO_2_ after alkali treatment	LOD: Cd 56.1 nM, Pb 16.5 nM, Cu 79.4 nM, Hg 56.7 nM	[181]
					Recovery rate: 96.0%–104.3%	
Mycotoxin	Peanut	AFB_1_	Portable NIR spectroscopy	IVSO initial screening, BWO optimization of feature variables	RMSE 24.6322 μg·kg^−1^, correlation coefficient 0.9761, relative percent deviation 4.6999	[182]
				SVM		
	Peanut	AFB_1_ and total aflatoxin	Short-wave infrared hyperspectral imaging (SWIR-HSI)	IVISSA-SPA-PLSR	Accurate prediction of AFB_1_ and total aflatoxin content	[183]
					Residual prediction deviation: 2.7959 and 2.7274	
					Limits of detection: 29.3722 and 45.7429 μg/kg	
	Oat	Deoxynivalenol (DON)	NIR-HSI	PLS	RMSEP 403.18 μg/kg, *R*^2^ 0.75	[184]
					The most contributing wavelengths are 1,203 and 1,388 nm	
	Maize	Zearalenone (ZEN)	MSI	GA-BPNN	The accuracy of ZEN pollution levels is 93.33%	[185]
					*R*_p_ 0.95, RMSEP 3.66 μg/kg, RPD 5.39, bias 1.55 μg/kg	
	Maize and wheat	DON	SERS	Designed a probe consisting of an AuNR@Ag core, an ultra-thin SiO_2_ layer and an AuNPs satellite with high surface coverage	LOD 0.053 fg/ml	[186]
					Wide linear range: 0.1 fg/ml to 1 μg/ml	
	Soybean oil	AFB_1_	THz	Signal pretreatment: *t* SNE	BPNN combined with t-SNE predicted the best results	[187]
				LS-SVM, BPNN, RF, and PLS	*R*_p_ 0.9948, RMSEP 0.7124 μg/kg	
	Maize	ZEN	Electrochemical sensor	ECL immunosensor was established using SnO_2_ QDs and Pd-GO	Wide linear range: 0.0005–500 ng/ml	[188]
					LOD 0.16 pg/ml	
					Recovery rate: 82.5%–96.3%, RSD < 12.8%	
	Maize	AFB_1_	E-nose	SVM and k-NN	Accuracy: 68%–94%	[189]

HSI, hyperspectral imaging; GC-MS, gas chromatography–mass spectrometry; PLS-DA, partial least squares discriminant analysis; LWPLSR, locally weighted partial least square regression; AWLS-GSD, automatic weighted least squares and gap segment derivative; RC, regression coefficient; SERS, surface-enhanced Raman spectroscopy; LOD, limit of detection; PCA, principal component analysis; LDA, linear discriminant analysis; SVM, support vector machine; NIR, near-infrared; PLSR, partial least squares regression; RMSEP, root mean square error of prediction; THz-TDS, terahertz time-domain spectroscopy; BIC, bound states in the continuum; RF, random forest; ELM, extreme learning machine; NMR, nuclear magnetic resonance; NIRS, near-infrared spectroscopy; CDELM, constrained difference extreme learning machine; RMSE, root mean squared error; MRE, mean relative error; PLS, partial least squares; AFB_1_, aflatoxin B_1_; IVSO, iteratively variable subset optimization; BWO, beluga whale optimization; IVISSA, interval variable iterative space shrinkage approach; SPA, sequential projection algorithm; MSI, multispectral imaging; LS-SVM, least squares support vector machine; BPNN, backpropagation neural network; QDs, quantum dots; k-NN, k-nearest neighbors

### Physical hazard detection

Physical hazards are the unintentional presence of foreign objects in food that can pose risks to consumers. The common physical hazards comprise insects, dust, glass fragments, metal shavings, wood splinters, plastic particles, and bones. These contaminants can cause mechanical injuries, such as cuts and punctures, and may also lead to choking or more severe health issues. Such contamination can occur at any stage of the food supply chain. Therefore, to prevent these incidents, manual screening methods can be used; however, they are time-consuming, labor-intensive, and often ineffective for detecting small foreign objects that are difficult to see with the naked eye.

To address these challenges, researchers have explored various technological methods to detect physical contaminants in food. For instance, grains stored for long periods in granaries, if not properly managed, may be subject to pest infestations. Pests can breach physical barriers and leave behind harmful foreign objects, such as excrement, carcasses, and other secretions, rendering the grains unfit for consumption. To address this issue, Biancolillo et al. [121] used combining NIRS with chemometric methods to develop PLS-DA and soft independent modeling of class analogy models, achieving over 90% accuracy in distinguishing between edible rice and insect-infested rice in test sets. In addition, Srivastava and Mishra [122] utilized Vis light and NIR hyperspectral technology to detect and differentiate *Sitophilus oryzae*, a common rice pest, from healthy rice. Applications of technologies such as NIR, HSI, THz, and NMR for detecting foreign objects in food are quite extensive [123,124]. For instance, spectroscopic detection technologies can identify contaminants by analyzing the unique spectral features of different materials at specific wavelengths. NMR works by exciting protons in food materials, and as these protons return to their ground state, they release energy that is converted into image data. The differences in the image data can then be used to identify foreign objects [125].

## Challenges and Solutions

### Accuracy and stability requirements

Intelligent sensing technology has substantial potential in food safety detection, although it faces several challenges. The complexity of food matrices hinders the accurate identification and quantification of target substances. Achieving a balance between detection sensitivity and specificity requires optimizing factors such as detection limits, chemical fingerprint recognition, complex food matrix handling, and operation in harsh environments, where the stability and adaptability of current technologies often fall short.

To address these challenges and enhance the role played by intelligent sensing technology in food safety monitoring, several strategies can be implemented. First, advancing research and development to improve the precision and reliability of sensing technologies is essential. As sensors are the core components of intelligent sensing systems, their performance directly impacts detection accuracy. Employing high-precision sensors combined with digital filtering techniques, such as moving average, median, and Kalman filtering [126–128], effectively reduces noise and interference, thereby boosting the signal-to-noise ratio and enhancing detection precision. Additionally, using compressed sensing techniques, as demonstrated by Fessler in NMR image reconstruction, enhances imaging velocity and image clarity while minimizing artifacts [129]. Moreover, these methods facilitate clinical applications by decreasing the number of detection operations, increasing efficiency, and reducing costs, yet maintaining high accuracy [130]. Integrating artificial intelligence and real-time big data mining into data analysis enhances the collection and integration of multisource heterogeneous data. The use of machine learning and deep learning algorithms greatly boosts the accuracy and efficiency of detection systems. Commonly employed machine learning techniques in food safety detection are Bayesian networks, neural networks, RF, and SVM [97]. Building on these advancements, the integration of multimodal feature technologies represents a further evolution in intelligent sensing, merging inputs from diverse sensory modalities like vision, olfaction, and taste to enhance the efficacy and accuracy of food safety detection systems.

Multimodal feature integration technology is expected to see further advancement by leveraging information from multiple sensory modalities, such as vision, olfaction, and taste, thereby providing a more comprehensive approach to food safety detection. Key fusion strategies include data-level, feature-level, and decision-level fusion [80]. Multimodal fusion, by offering more complete and accurate information than single-data analysis, enhances decision-making intelligence, automation, and model robustness [131–133]. For instance, Li et al. [134] applied Vis-NIR and NIR-HSI systems along with data fusion technology to predict enrofloxacin residue levels in lamb, demonstrating that data fusion markedly outperformed single-source data, particularly at the feature-level fusion ($R_{P}^{2}$ = 0.940, RMSEP = 0.375). Similarly, Shen et al. [135] integrated Vis-HSI with MV technologies for real-time detection of *Aspergillus* spp. and *Fusarium* spp. in stored corn. Through LDA-based data fusion, the combined approach achieved a 5.5% accuracy improvement in fungal strain differentiation and contamination level classification compared to unimodal methods. Through the integration of various sensor types and data sources, multimodal fusion technology greatly enhances the accuracy, efficiency, and scope of food safety detection. Further advancements in sensor technology are crucial, as they build on how multimodal fusion technology enhances food safety detection via comprehensive data analysis. These innovations focus not only on integrating multiple sensing modalities but also on refining the capabilities of individual sensors to resist interference and improve overall system reliability.

Developing new sensor technologies to enhance anti-interference capabilities is essential for advancing food safety detection. This development hinges on selecting advanced sensing materials and applying cutting-edge techniques, such as bio- and electrochemical methods. These approaches enhance the sensitivity, specificity, stability, and anti-interference performance of sensors. Metal-organic frameworks, noted for their orderly structure, high surface area, and permanent porosity, are widely used for detecting mycotoxins [136], heavy metals [137], and pathogens [138]. Advances in aptamer screening technologies have notably enhanced the specificity and applicability of detecting heavy metal ions in complex grain matrices, overcoming the challenge of obtaining heavy metal antibodies in grains [139]. Furthermore, microfluidic electrokinetic effects facilitate efficient target enrichment of heavy metals in food, with nucleic acid aptamers and other probes providing selective recognition [140]. Nanocolorimetric sensing technology is especially effective in detecting mold in food and grain crops, overcoming the limitations faced by traditional volatile substance detection sensors, such as MOS-type gas sensors, which suffer from poor stability and environmental sensitivity [141].

### Industrial integration and digital transformation

In the complex landscape of global food safety regulation, the integration of intelligent sensing technology is driving innovation in the food industry. By mimicking human sensory organs, intelligent sensors can capture various sensory attributes of food samples. When combined with intelligent recognition algorithms for signal processing, these sensors allow a comprehensive, human-like assessment of food quality. As Kwon [142] has noted, intelligent sensing technology can transform sensory data into big data that support personalized dietary decisions, catering to the growing consumer demand for customized food. This advancement represents a crucial direction for the food industry during the Fourth Industrial Revolution.

Leveraging digital technology to integrate food information with advanced technologies has led to the creation of precise digital food products. The food industry is evolving into a new industrial model through the integration of digital technology and physical enterprises, promoting sustainable development. To achieve end-to-end quality control, digital technology enables full traceability of food from production to distribution, sale, and consumption. However, traditional traceability systems often face challenges such as fragmented data storage and vulnerability to tampering. This is where decentralized technologies like blockchain become critical: As an immutable digital ledger, blockchain enhances transaction transparency and food safety by providing a unified platform for recording and verifying every step of the supply chain [143]. Consumers can access comprehensive information about a product’s origin, processing journey, and the path it took to reach store shelves. This helps build trust and serves as a safeguard against fraudulent activities.

Artificial intelligence (AI) and Internet of Things (IoT) technologies synergize with blockchain to enhance analytical accuracy and real-time oversight. Liu et al. [144] proposed an integrated AI–IoT–blockchain framework that leverages spectroscopy, mass spectrometry, visual imaging, and sensor networks combined with machine/deep learning algorithms. This integration facilitates rapid adulteration detection, predictive quality management, and automated fraud identification. For example, IoT sensors deployed on beverage production lines can monitor parameters such as mixing consistency, bottle-filling precision, and packaging integrity, while blockchain immutably logs these data to ensure accountability across manufacturing stages [145]. Zhang et al. [146] developed flexible paper-based sensors capable of tracking critical variables like moisture, humidity, and fruit weight during storage and transportation. These sensors transmit real-time data to smartphones, enabling proactive interventions to mitigate spoilage risks. Environmental factors, such as temperature, humidity, and oxygen levels, are continuously monitored through blockchain-linked smart sensors, generating tamper-evident records of storage and transport conditions. This dual-layer architecture, combining IoT for real-time data acquisition and blockchain for secure verification, not only safeguards food safety but also reduces operational costs. Additionally, blockchain-powered smart contracts automate compliance checks and transaction processes. For instance, a beverage manufacturer can encode quality assurance protocols into self-executing contracts, which automatically validate batch-specific data across distributed nodes. This automation streamlines supply chain operations while providing consumers with cryptographically verifiable product information, thereby reinforcing trust [147].

### Energy efficiency requirements

Digitalization is driving the transition to green development by accelerating the integration of digital and environmental technologies. Intelligent sensing technology reduces resource consumption and environmental pollution during detection while enhancing production efficiency and product quality through intelligent manufacturing. In addition, it contributes to lower energy consumption and carbon emissions. Utilizing sensors, artificial intelligence, and big data analysis, intelligent sensing technology can rapidly and accurately detect harmful substances in food, thereby safeguarding consumer health [144]. Moreover, it promotes resource recycling, improves energy efficiency, strengthens environmental impact assessments, and supports the development of green supply chains. The integration of blockchain technology further enhances transparency and efficiency within the food supply chain, addressing issues such as information sharing and procedural irregularities [145]. Collectively, the application of these technologies not only ensures food safety and quality but also promotes resource conservation and environmental protection, providing strong support for the greening and sustainable development of the food industry.

To further enhance energy efficiency, intelligent sensing technology should prioritize low-power sensor designs and optimized AI algorithms that minimize computational overhead, ensuring sustainable operation over long periods. This principle has been successfully demonstrated in recent innovations. Researchers at Koç University in Istanbul, Turkey have developed a miniature sensor that can monitor food freshness in real time wirelessly and battery-free, while transmitting results to smartphones. The sensor is created by layering an easily synthesized polymer onto electrodes, utilizing capacitive sensing technology to detect biogenic amines produced by protein-rich foods. Weighing approximately 2 g with dimensions of 0.3 inches (2 cm^2^), the sensor employs near-field communication (NFC) technology. Its chip connects to smartphones through an antenna, enabling real-time wireless data transmission. When an NFC-enabled smartphone is brought near the sensor, the chip harvests sufficient energy through proximity-based power transfer [148]. While energy-efficient hardware design addresses data acquisition challenges, intelligent data processing in food safety systems requires complementary algorithmic optimization. These demands become particularly acute when processing complex image data, which necessitates substantial hardware investments and incurs considerable energy consumption. To mitigate these dual challenges, researchers are adopting a threefold approach: long-term strategic investments amortize upfront costs across extended operational cycles, while cloud-edge computing integration allocates computationally intensive model training to centralized cloud infrastructure and delegates latency-sensitive inference tasks to decentralized edge devices. Furthermore, advanced model compression techniques, such as quantization and pruning, dramatically lower memory footprints and energy requirements without compromising detection accuracy. This synergistic framework not only enhances computational sustainability but also enables real-time, energy-conscious food safety monitoring at scale [149].

## Conclusion

This review analyzes the application of intelligent sensing technologies in food safety fields, particularly their remarkable progress in identifying microbial, chemical, and physical hazards in food. It delves into the fundamental principles of technologies such as optical detection, electrochemical detection, machine olfaction, and machine gustation. Through the synergy of multisource sensors and smart computing, the improvements made by these technologies to traditional detection methods are not limited to the enhancement of detection accuracy and efficiency. Moreover, the detection accuracy and limitation of relevant indicators must meet food safety standards while ensuring stability and speed. The applications of intelligent sensing technologies can be applied across various sectors of the modern food industry. Integrating with machine learning and blockchain, it can optimize processing technology, assist decision-making, and minimize losses throughout all food industry chain. Despite the remarkable advancements in intelligent sensing technologies, several challenges remain, such as interference from complex food matrices, the demand for real-time detection, and the integration of large-scale data processing.

With the continuous advancement of sensor resolution and algorithmic robustness, emerging intelligent sensing systems will bring more transformative changes to the food industry especially for food safety. Overall, these innovations are crucial for enhancing global food security and sustainability, ensuring a safer, more transparent, and resilient food supply chain that meets the evolving demands of consumers and regulatory bodies.
